# Growth Outcomes and Relapse Risk in Pediatric Medulloblastoma Survivors with and Without Growth Hormone Therapy: A 23-Year Single-Center Cohort Study

**DOI:** 10.3390/jcm15093472

**Published:** 2026-05-01

**Authors:** Gerdi Tuli, Jessica Munarin, Paola Ragazzi, Eleonora Biasin, Francesco Felicetti, Anna Mussano, Stefano Gabriele Vallero, Daniele Bertin, Paola Peretta, Giovanni Morana, Franca Fagioli, Luisa De Sanctis

**Affiliations:** 1 Department of Pediatric Endocrinology, Regina Margherita Children’s Hospital, 10126 Turin, Italy; jessica.munarin@unito.it (J.M.); luisa.desanctis@unito.it (L.D.S.); 2 Department of Pediatric and Public Health Sciences, University of Turin, 10124 Turin, Italy; eleonora.biasin@unito.it (E.B.); stefano.vallero@unito.it (S.G.V.); daniele.bertin@unito.it (D.B.); franca.fagioli@unito.it (F.F.); 3 Department of Pediatric Neurosurgery, Regina Margherita Children’s Hospital, 10126 Turin, Italy; pragazzi@cittadellasalute.to.it (P.R.); pperetta@cittadellasalute.to.it (P.P.); 4 Pediatric Onco-Hematology, Stem Cell Transplantation and Cellular Division, Regina Margherita Children’s Hospital, 10126 Turin, Italy; 5 Oncological Endocrinology, Department of Oncology, AOU Città Della Salute e Della Scienza, 10126 Turin, Italy; francesco.felicetti@unito.it; 6 Radiotherapy Unit, Regina Margherita Children’s Hospital, 10126 Turin, Italy; anna.mussano@unito.it; 7 Neuroradiology Unit, AOU Città Della Salute e Della Scienza, 10126 Turin, Italy; giovanni.morana@unito.it; 8 Department of Neurosciences, University of Turin, 10126 Turin, Italy

**Keywords:** medulloblastoma, growth hormone deficiency, pediatric age, children, growth outcome

## Abstract

**Background:** Growth hormone deficiency (GHD) is one of the most common endocrine sequelae in survivors of pediatric medulloblastoma, largely resulting from hypothalamic–pituitary irradiation. Concerns regarding the oncologic safety of growth hormone (GH) replacement have historically limited its use. This study aimed to evaluate growth response to GH therapy and its potential association with tumor relapse in medulloblastoma survivors treated between 2000 and 2023. **Methods:** We conducted a retrospective single-center cohort study including 74 patients diagnosed with medulloblastoma before 18 years of age. GHD was confirmed by stimulation testing according to standard criteria. Auxological, endocrine, and oncologic data were collected longitudinally. Growth outcomes were compared among patients without GHD (*n* = 38), patients with untreated GHD (*n* = 13), and patients with GHD receiving GH treatment (*n* = 23). Relapse rates were assessed following GH initiation and compared with those of untreated patients. **Results:** GHD was diagnosed in 48.7% of patients. Baseline height SDS did not differ among groups. Patients with untreated GHD experienced a significant decline in height SDS (−1.93 ± 0.78), whereas GH-treated patients showed a significant increase (+0.39 ± 0.06; *p* < 0.0001). Final height SDS was significantly lower in untreated GHD patients (−2.45 ± 0.36) compared with GH-treated patients (−1.71 ± 0.68) and patients without GHD (−0.68 ± 0.24; *p* < 0.0001). No evidence of an increased risk of tumor relapse was observed in association with GH therapy during follow-up. **Conclusions:** GH replacement significantly improves growth outcomes in medulloblastoma survivors with confirmed GHD without apparent increase in relapse risk when initiated after stable remission. The early identification and multidisciplinary management of GHD are essential components of long-term survivorship care.

## 1. Introduction

Paediatric medulloblastoma is a primitive neuroectodermal tumor, mostly located in the posterior fossa with a wide differential diagnosis for lesions in that location for that specific group age, accounting for approximately 15–20% of all pediatric central nervous system (CNS) malignancies [1]. Over the past several decades, major advances in neurosurgical techniques, craniospinal irradiation protocols, chemotherapy regimens, and risk-adapted treatment strategies have led to substantial improvements in survival [2,3,4,5,6]. Contemporary multimodal approaches now achieve long-term overall survival rates exceeding 70–80% in many standard-risk and selected high-risk patient groups [2,3,4,5]. In parallel, the introduction of molecular subgrouping has refined prognostication and informed treatment de-escalation strategies aimed at preserving efficacy while minimizing long-term toxicity [6].

As survival rates have improved, the population of long-term medulloblastoma survivors has steadily increased, shifting clinical focus toward survivorship and the prevention, early detection, and management of late effects [7,8,9,10,11]. Survivors frequently experience a broad spectrum of chronic health conditions, including neurocognitive impairment, sensory deficits, musculoskeletal complications, and endocrine dysfunction, collectively imposing a substantial burden on quality of life and long-term health [7,8,9,10]. Endocrine sequelae are particularly prevalent and clinically impactful, often requiring lifelong surveillance and intervention [11].

Damage to the hypothalamic–pituitary axis represents a central mechanism underlying endocrine morbidity in medulloblastoma survivors and is primarily attributable to craniospinal irradiation, although surgery and chemotherapy may also contribute [12,13,14,15,16]. Among endocrine disorders, growth hormone deficiency (GHD) is the most common and typically the earliest to manifest following treatment [17,18,19,20,21,22,23]. Its prevalence increases over time and is strongly associated with radiation dose to the hypothalamus and pituitary, younger age at treatment, and cumulative exposure to multimodal therapy [12,17,18,19,20]. Even relatively low radiation doses to the hypothalamic–pituitary region can impair GH secretion, underscoring the particular vulnerability of this axis in pediatric patients [13,16].

Longitudinal cohort studies have demonstrated that a substantial proportion of medulloblastoma survivors initially develop isolated GHD, with a risk of progression into multiple pituitary hormone deficiencies over time [21,22,23,24,25,26,27,28]. The clinical consequences of untreated GHD extend beyond impaired linear growth. Affected survivors may experience reduced final adult height, unfavorable alterations in body composition with increased adiposity and reduced lean mass, decreased bone mineral density, dyslipidemia, insulin resistance, and diminished health-related quality of life [29,30,31,32,33,34,35]. These sequelae are particularly concerning given the already elevated cardiometabolic risk observed in childhood cancer survivors.

Recombinant human growth hormone (rhGH) replacement therapy has been shown to effectively address many of these adverse outcomes in GH-deficient survivors of pediatric medulloblastoma. Multiple studies have demonstrated improvements in growth velocity and final adult height, especially when treatment is initiated promptly after confirmation of GHD and before epiphyseal closure [2,16,21,29,30,31,32]. Treatment response may vary according to factors such as age at GH initiation, radiation field and dose, and the extent of spinal irradiation, highlighting the importance of individualized treatment strategies [16].

Despite its demonstrated benefits, GH replacement therapy in survivors of CNS tumors has historically been approached with caution. Concerns stemmed primarily from the biological plausibility that activation of the GH–IGF-1 (Insulin-like Growth Factor 1) axis could stimulate residual tumor cells or promote tumor recurrence, progression, or the development of secondary neoplasms [32,36,37,38,39]. Early reports and small observational studies raised apprehension regarding GH safety, resulting in delayed initiation or avoidance of therapy during critical periods of growth and development [40,41].

Over the past two decades, however, a robust body of evidence has accumulated to address these concerns. Data from cohort studies, registry analyses, systematic reviews, and international growth hormone surveillance databases consistently indicate that GH replacement therapy does not increase the risk of medulloblastoma recurrence or progression when initiated after completion of oncologic treatment and in the context of radiologically stable disease [42,43,44,45,46,47]. Importantly, large-scale analyses suggest that the incidence of secondary neoplasms in GH-treated survivors is more closely related to prior exposure to craniospinal irradiation than to GH therapy itself [3,12,29,38,39,41,46].

Concurrently, advances in medulloblastoma treatment strategies have sought to reduce long-term toxicity while maintaining oncologic efficacy. Reduced-dose craniospinal irradiation, posterior fossa-sparing approaches, and the increasing adoption of proton beam therapy have been associated with improved normal tissue sparing and may mitigate, though not eliminate, endocrine morbidity [48,49,50,51,52,53]. Emerging evidence indicates that while proton therapy may reduce radiation exposure to the hypothalamic–pituitary axis, clinically significant endocrine dysfunction—including GHD—remains common, reinforcing the need for vigilant long-term endocrine follow-up [13,14,16,18,19,20].

Within this evolving therapeutic landscape, growth hormone replacement therapy has become an increasingly accepted and integral component of comprehensive survivorship care for pediatric medulloblastoma survivors. Current expert consensus statements and clinical practice guidelines emphasize careful patient selection, appropriate timing of therapy initiation, coordination with oncology teams, and long-term surveillance to ensure both efficacy and safety [24,25,42,43,47].

The present study aims to evaluate the efficacy and oncological safety of growth hormone replacement therapy in pediatric medulloblastoma survivors followed at a tertiary care center over the period between 2000 and 2023, with particular attention paid to growth outcomes and oncological relapse risk.

## 2. Materials and Methods

### 2.1. Study Design and Population

This retrospective cohort study included pediatric patients diagnosed with medulloblastoma between January 2000 and December 2023 at Regina Margherita Children’s Hospital. Patients were identified through institutional neuro-oncology and pediatric endocrinology databases. All patients with a confirmed diagnosis of medulloblastoma underwent a complete baseline endocrine assessment at diagnosis, followed by evaluations every six months in the presence of endocrine impairment or annually if no endocrine disorder was detected, as detailed in our previous paper [28]. The study was conducted in accordance with the **Declaration of Helsinki** and approved by the Institutional Review Board of The Health and Science University Hospital of Turin (Protocol No. 1345/A). Written informed consent for the use of anonymized clinical data was obtained from all patients and/or their legal guardians.

### 2.2. Eligibility Criteria

Eligible patients had a histologically confirmed diagnosis of medulloblastoma according to World Health Organization criteria, were diagnosed at ≤18 years of age, and had completed oncologic treatment, including surgery with or without radiotherapy and chemotherapy. Growth hormone deficiency had to be formally diagnosed during follow-up, and sufficient auxological, endocrine, and oncologic data had to be available for longitudinal evaluation. Patients were required to have a minimum follow-up duration of one year after initiation of GH therapy or, for untreated patients, after the diagnosis of GHD. Patients were excluded if follow-up data were insufficient, or if they had genetic or congenital conditions independently associated with growth or pituitary dysfunction.

### 2.3. Data Collection and Clinical Variables

All patients with a confirmed diagnosis of medulloblastoma underwent regular endocrine baseline assessment at diagnosis and thereafter every six months if any endocrine impairment was found or annually if there was not any endocrine disorder. Endocrine follow-up started at the diagnosis and lasted until the transition age. Collected variables included demographic characteristics, age at medulloblastoma diagnosis, and sex. Detailed treatment-related information was recorded, including extent of surgical resection, use and dose of craniospinal irradiation, radiation boost fields, and chemotherapy protocols. Endocrine data included age at diagnosis of growth hormone deficiency, results of GH stimulation testing, serum IGF-1 levels, presence of additional pituitary hormone deficiencies, and longitudinal growth measurements. GH treatment variables included age at GH initiation, dosage adjustments over time, treatment duration, and biochemical monitoring. Final height was defined as the height achieved when growth velocity was <2 cm/year and epiphyseal closure was reached. Oncologic outcomes, including tumor relapse or progression, secondary neoplasms, and survival status, were systematically recorded, along with dates of relevant events and duration of follow-up.

### 2.4. Diagnosis of Growth Hormone Deficiency

All children presenting growth impairment during the endocrine follow-up underwent stimulation testing for growth hormone secretion assessment. Growth hormone deficiency was diagnosed according to international pediatric endocrinology guidelines. Diagnosis was based on impaired linear growth or reduced height velocity in combination with biochemical evidence of insufficient GH secretion. Biochemical confirmation required a subnormal peak GH response on at least two pharmacological stimulation tests (arginine and glucagon), using assay-specific cut-off values (GH < 8 mcg/L after two different stimulation tests). Patients with multiple pituitary hormone deficiencies underwent comprehensive endocrine evaluation and received appropriate hormonal replacement as clinically indicated.

### 2.5. Growth Hormone Treatment Protocol

Recombinant human growth hormone was prescribed in accordance with local clinical practice and international recommendations. Treatment was initiated only after confirmation of stable oncologic disease for at least two years, following multidisciplinary discussion between pediatric endocrinologists and oncologists, and after obtaining parental consent. GH was administered as a daily subcutaneous injection, with initial dosing based on body weight and subsequent titration aimed at optimizing growth response while maintaining serum IGF-1 concentrations within the normal age- and sex-adjusted reference range (0.15–0.25 mg/kg/week). Patients were monitored regularly for treatment efficacy, adherence, and potential adverse effects.

### 2.6. Outcome Measures

The primary efficacy outcome was the response to GH therapy, assessed by changes in height standard deviation score from the start of GH treatment to the last available follow-up and by annualized height velocity during treatment. Secondary growth-related outcomes included the proportion of patients achieving near-adult height within the normal population range and the temporal pattern of growth response. The primary safety outcome was tumor relapse or progression following GH initiation. Relapse was defined as radiologically or histologically confirmed recurrence of medulloblastoma after a documented period of remission. Time to relapse was calculated from the date of GH initiation for treated patients and from a corresponding reference time point for untreated GHD patients.

### 2.7. Comparative Analysis and Control Group

To evaluate the potential association between GH therapy and relapse risk, outcomes in GH-treated patients were compared with those in two control groups, i.e., medulloblastoma survivors with confirmed GHD who did not receive GH therapy and survivors without GHD. The three groups were comparable with respect to age at diagnosis, sex, treatment era, radiation exposure, and duration of follow-up. Where appropriate, matching techniques and multivariable adjustment were applied to minimize confounding related to disease severity and treatment characteristics.

### 2.8. Statistical Analysis

Descriptive statistics were used to summarize baseline characteristics and treatment variables. Continuous data were reported as means with standard deviations (SDs) or medians with interquartile (IQR) ranges, while categorical variables were expressed as counts and percentages. Relapse-free survival was analyzed using Kaplan–Meier methods, with comparisons between groups assessed by log-rank testing. Cox proportional hazards regression models were used to estimate hazard ratios for relapse associated with GH therapy. Statistical significance was defined as a two-sided *p* value of less than 0.05. Statistical analyses and graphs were performed using GraphPad 7 software (GraphPad Software, La Jolla, CA, USA).

## 3. Results

### 3.1. Patient Characteristics

A total of 74 patients with a history of medulloblastoma were included in the analysis (Table 1). The cohort comprised 35 males (47.3%) and 39 females (52.7%), with a mean age at diagnosis of 7.94 ± 0.56 years and a median age of 8 years (IQR 5–11). The mean follow-up duration was 12.01 ± 0.52 years with a median of 10 years (IQR 9.4–15).

Nearly all patients underwent surgical resection (95.9%), and the majority received chemotherapy (94.6%) and radiotherapy (90.5%) as part of their oncologic management. Photon-therapy was administered to 55 patients, whereas 8 patients received proton therapy. The mean radiation dose to the tumour bed was 44.1 ± 12.01 Gy, while the mean craniospinal irradiation dose was 30.5 ± 8.01 Gy. Most patients receiving radiotherapy were treated to both the tumour bed and the craniospinal axis (62/67, 92.5%), whereas 5/67 (7.5%) received irradiation to the tumour bed only. Eight patients were classified as Infant High Risk, while seven patients did not receive radiotherapy. Molecular subgroup data were available for 20 patients. The majority (13/20, 65%) showed no activation of Sonic Hedgehog (SHH) or WNT pathways and no TP53 mutation. Four patients (4/20, 20%) exhibited both SHH activation and TP53 mutation, while two (2/20, 10%) showed isolated SHH activation. Only one patient (1/20, 5%) demonstrated isolated WNT activation.

Endocrine disorders were documented in 64 patients (86.5%), while 10 patients (13.5%) did not develop endocrine complications during follow-up. Growth hormone deficiency was identified in 36 patients (48.7%). Among these, 23 patients (31.1% of the total cohort) received GH replacement therapy, whereas 13 patients (17.6%) remained untreated despite meeting endocrine and oncological indications, owing to the absence of parental consent. The remaining 38 patients (51.3%) did not meet the diagnostic criteria for GHD. No significant differences were observed among the three groups with respect to age at diagnosis and treatment regimen.

### 3.2. Baseline Auxological and Endocrine Characteristics

No statistically significant difference was observed regarding age at medulloblastoma diagnosis, baseline height Standard Deviation Score (SDS) and radiotherapy dose among the three groups (no GHD, GHD untreated, and GHD treated). The mean baseline height SDS in the overall cohort was −2.2 ± 0.73 (*p* = 0.85), as shown in Table 2.

No statistically significant difference was observed regarding age at GHD stimulation test and pubertal stages among the three groups (no GHD, GHD untreated, and GHD treated). Peak GH responses to stimulation tests were significantly reduced in both GHD groups compared with patients without GHD (20/38 patients were tested for GHD according to anthropometric criteria). After arginine stimulation, the overall mean GH peak was 6.4 ± 0.73 ng/mL, with significantly lower values in the untreated GHD group (1.83 ± 0.48 ng/mL) and the treated GHD groups (2.36 ± 0.48 ng/mL) compared with the no-GHD group (9.4 ± 1.01 ng/mL; *p* < 0.0001). Similar findings were observed after glucagon stimulation, with mean GH peaks of 2.52 ± 0.66 ng/mL and 2.67 ± 0.56 ng/mL in the untreated and treated GHD groups, respectively, compared with 10.3 ± 0.18 ng/mL in the no-GHD group (*p* < 0.0001).

The difference between chronological age and bone age (CA–BA) tended to be greater in the treated GHD group (3.49 ± 0.21 years) than in the untreated GHD group (2.23 ± 0.19 years), although this difference did not reach statistical significance (*p* = 0.07).

We did not observe differences in other pituitary axis impairments or endocrine dysfunctions. However, TSH deficiency and primary hypothyroidism appeared to be more frequent in patients with GHD, regardless of treatment status.

### 3.3. Growth Hormone Treatment Characteristics

GH treatment was initiated at a median of 3 years (IQR 6.75–10) after complete remission or the documentation of stable residual disease on neuroimaging. Among treated patients, the mean duration of GH therapy was 6.01 ± 3.24 years with a median of 6.5 years (IQR 3.75–8). Mean GH dose was 0.17 ± 0.01 mg/kg/week. Mean IGF-1 levels during follow-up were significantly higher in GH-treated patients (366.8 ± 27.12 ng/mL) than in untreated GHD patients (156.6 ± 25.56 ng/mL), while patients without GHD showed the highest mean IGF-1 levels (441.4 ± 120.9 ng/mL) (*p* < 0.0001).

### 3.4. Height Outcomes

**Figure 1:**
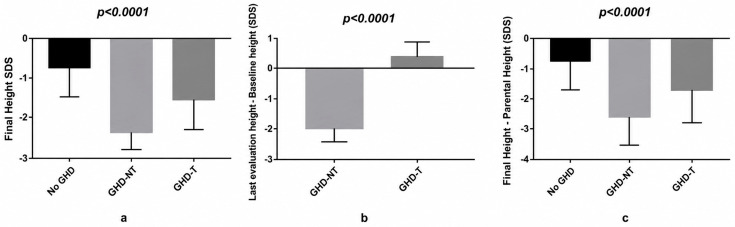
Differences in mean ± SD final height SDS (**a**), change in mean ± SD height SDS from baseline to last evaluation (**b**) and the difference between mean ± SD final height SDS and parental height (**c**) among the patients without GHD (no-GHD), with untreated-GHD (GHD-NT) and treated GHD (GHD-T).

At last evaluation, the mean height SDS in the overall cohort was −1.27 ± 0.22. As shown in Figure 1, significant differences were observed among groups (*p* < 0.0001). Patients without GHD had the highest height SDS (−0.78 ± 0.56), while GHD untreated patients had the lowest (−1.97 ± 0.42). GH-treated patients showed intermediate height SDS of −1.72 ± 0.32. The change in height SDS from baseline to last evaluation differed significantly among groups (*p* < 0.0001). Patients without GHD demonstrated a mean increase of 0.59 ± 0.26 SDS, while GH-treated patients demonstrated a positive gain of 0.39 ± 0.06 SDS. In contrast, untreated GHD patients experienced a marked decline (−1.93 ± 0.78 SDS), indicating progressive growth impairment in the absence of GH replacement.

Final height SDS also differed significantly among groups (*p* < 0.0001). The no-GHD group achieved the highest final height (−0.68 ± 0.24 SDS), followed by GH-treated patients (−1.71 ± 0.68 SDS), whereas untreated GHD patients had the lowest final height (−2.45 ± 0.36 SDS). Parental height SDS did not differ significantly among groups (*p* = 0.53), indicating comparable genetic height potential. However, the difference between final height SDS and parental height SDS was significantly greater in untreated GHD patients (−2.57 ± 0.33 SDS) than GH-treated patients (−1.73 ± 0.47 SDS) and patients without GHD (−0.77 ± 0.22 SDS) (*p* < 0.0001).

### 3.5. Relapse-Free Survival Analysis

During a median follow-up of 8–9 years, 9 relapse events were observed in the overall cohort (12.2%). Relapse occurred in 8.7% (2/32) of GH-treated patients, 15.4% (2/13) of untreated GHD patients, and 13.2% of patients without GHD (Table 3). The lowest crude relapse rate was observed in GH-treated patients.

**Figure 2:**
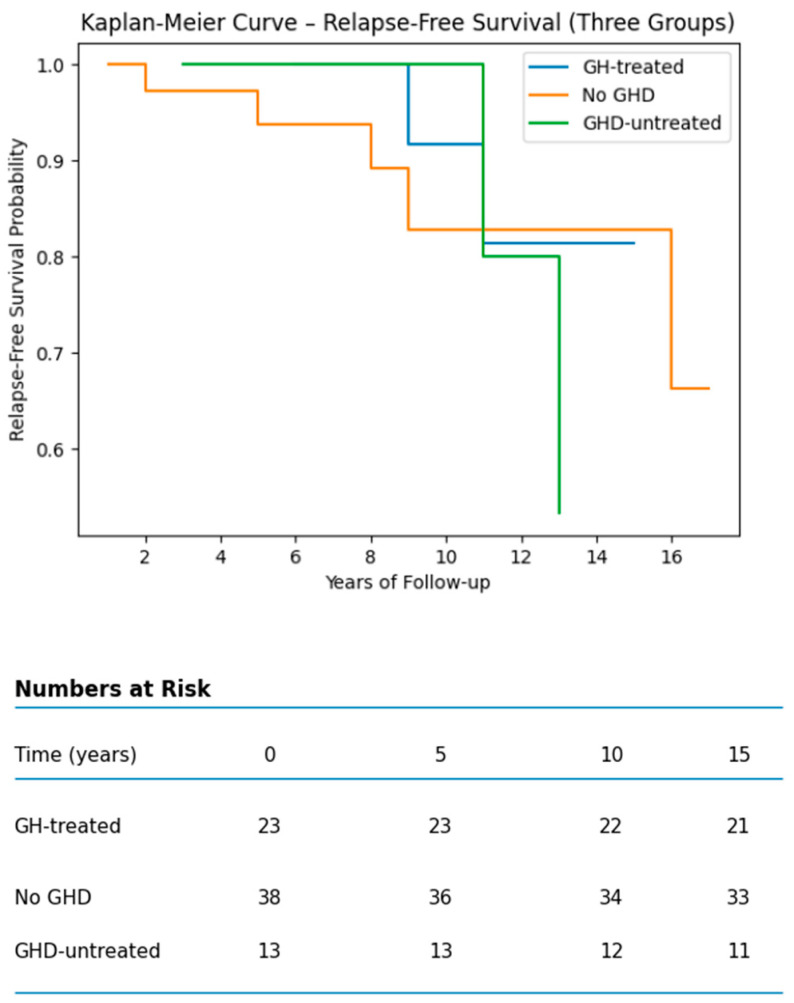
Kaplan–Meier curves of relapse-free survival in medulloblastoma survivors stratified by GH treatment status.

Kaplan–Meier curves for relapse-free survival (RFS) showed no statistically significant difference among the three groups (Figure 2). No statistically significant difference was observed between GH-treated and untreated GHD patients (log-rank *p* = 0.79).

At approximately 10 years, recurrence-free survival (RFS) was similar across groups: around 82% in growth hormone (GH)-treated patients, approximately 80% in patients with growth hormone deficiency (GHD) who were not treated, and about 83% in patients without GHD. The survival curves demonstrated overlapping trajectories without evidence of early relapse clustering following GH initiation. Cox proportional hazards analysis showed no significant association between GH treatment and relapse risk (HR 0.61, 95% CI 0.12–3.08; *p* = 0.55).

**Figure 3:**
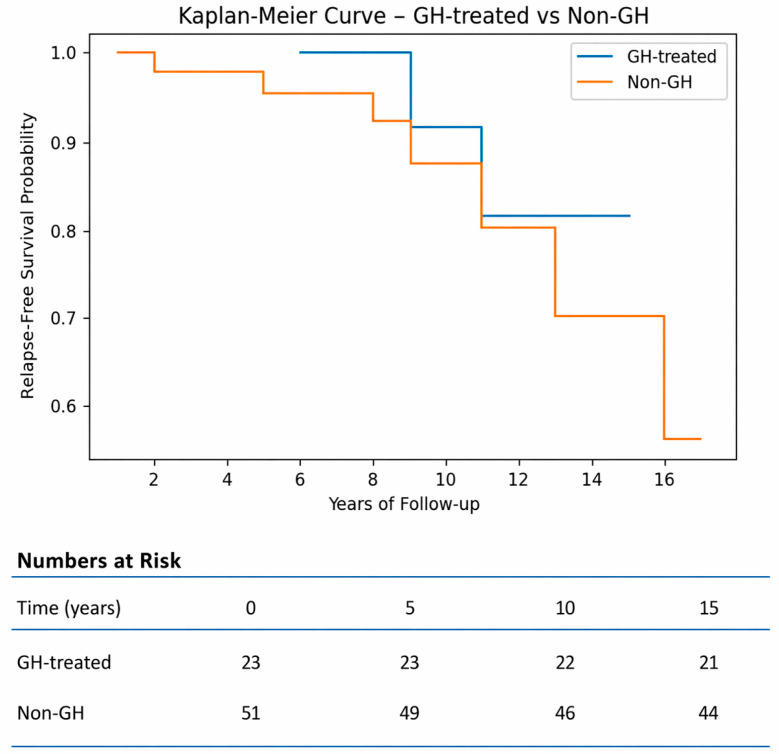
Kaplan–Meier curve comparing relapse-free survival between GH-treated patients and all non-GH treated patients.

Kaplan–Meier curves for relapse-free survival (RFS) showed no statistically significant difference between the patients treated with GH and the untreated patients (with and without GHD), as shown in Figure 3.

## 4. Discussion

The present study provides longitudinal evidence on the efficacy and oncologic safety of growth hormone (GH) replacement therapy in childhood survivors of medulloblastoma diagnosed between 2000 and 2023. In this cohort of 74 patients with a mean follow-up exceeding 12 years, nearly half developed confirmed growth hormone deficiency (GHD), and approximately one-third of the overall cohort received GH therapy. Our findings demonstrate that GH-treated patients achieved a significant improvement in height standard deviation score (SDS) compared with untreated GHD patients, with no evidence suggesting an adverse oncologic impact of GH therapy.

### 4.1. Prevalence of Endocrine Sequelae and Growth Hormone Deficiency

Consistent with previous reports, endocrine morbidity was highly prevalent in our cohort, affecting 86.5% of survivors. This observation aligns with prior studies demonstrating that hypothalamic–pituitary dysfunction is among the most common late effects in medulloblastoma survivors, particularly following craniospinal irradiation [12,13,14,15,16,17,18,19,20,21,22,23]. Growth hormone deficiency represented the most frequent endocrine disorder, affecting nearly half of patients, comparable to previously reported rates ranging from 40% to 80% depending on radiation dose, follow-up duration, and testing methodology [13,18,22]. The markedly reduced GH peak responses to arginine and glucagon stimulation in both treated and untreated GHD groups confirm the robustness of the biochemical diagnosis. As shown in prior dose–response analyses, GH secretion is particularly sensitive even to moderate hypothalamic radiation exposure [12], and dysfunction may progress over time [21,22,23,24,28]. The absence of significant baseline height SDS differences among groups further supports that post-treatment growth trajectories, rather than initial stature, determined final auxological outcomes. Hypothalamic–pituitary dysfunction and the evaluated endocrine morbidities did not differ among the three groups, with the exception of TSH deficiency and primary hypothyroidism, which were more frequent in patients with GHD regardless of GH treatment. However, this difference did not influence the response to stimulation testing or to therapy, as thyroid hormone replacement was promptly initiated in all hypothyroid patients.

### 4.2. Growth Outcomes and Efficacy of GH Therapy

The central finding of this study is the significant positive change in height SDS observed in GH-treated patients, in contrast to the progressive decline seen in untreated GHD patients. Although treated individuals did not fully normalize height SDS compared with patients without GHD, they achieved a clinically meaningful recovery of growth velocity and improved final height relative to untreated counterparts. These findings are consistent with previous reports demonstrating improved height velocity and final adult height in GH-treated medulloblastoma survivors [2,16,21,29,30,31,32]. Long-term follow-up studies have shown that early initiation of GH therapy after confirmation of disease stability optimizes growth response, particularly before advanced skeletal maturation [29,30]. The mean duration of GH therapy in our cohort (approximately six years) is comparable to durations reported in registry-based analyses and single-center series [2,45]. Importantly, untreated GHD patients experienced a significant decline in height SDS and the greatest negative deviation from parental height SDS, underscoring the progressive nature of radiation-induced GHD when left untreated. This observation mirrors findings from longitudinal studies demonstrating that failure to initiate GH therapy may result in severe adult short stature and long-term metabolic consequences [22,24,32]. Although GH-treated patients did not reach the same final height as those without GHD, this is expected given the multifactorial growth impairment associated with spinal irradiation, chemotherapy, and skeletal damage [34,35]. Spinal growth restriction after craniospinal irradiation remains a major determinant of final height, even in the presence of adequate GH replacement [34].

### 4.3. IGF-1 Dynamics and Treatment Adequacy

Mean IGF-1 levels during follow-up were significantly higher in GH-treated patients than in untreated GHD patients, confirming treatment adherence and biological efficacy. Although IGF-1 levels remained lower than those observed in patients without GHD, they were maintained within acceptable therapeutic ranges. This finding is particularly relevant in light of prior concerns regarding potential oncogenic effects associated with elevated IGF-1 levels [32,36,37,38,39]. In our cohort, no evidence suggested sustained supraphysiological exposure.

### 4.4. Oncologic Safety and Relapse Risk

A primary concern surrounding GH therapy in survivors of CNS malignancies has been the theoretical risk of tumor recurrence or progression due to the mitogenic properties of the GH–IGF-1 axis [32,36,37,38,39]. However, no signal of increased risk was observed in our limited cohort of medulloblastoma survivors, in line with accumulating evidence suggesting that GH replacement does not appear to increase the risk of recurrence after confirmed remission or stable residual disease. Nevertheless, our study was underpowered to provide definitive conclusions on oncological safety. Multiple large-scale cohort studies and international consensus statements have consistently demonstrated no significant association between GH therapy and medulloblastoma relapse when initiated after adequate disease-free intervals [42,43,44,45,46,47]. Registry-based analyses, including long-term surveillance data, have similarly failed to demonstrate increased tumor recurrence rates attributable to GH therapy [29,39,41,46]. Moreover, several investigations indicate that secondary neoplasm risk is primarily driven by prior radiotherapy exposure rather than GH treatment itself [3,12,38,39]. Kaplan–Meier analyses in our cohort showed no increased relapse risk associated with GH therapy. Relapse incidence was numerically lower in treated patients compared to untreated GHD patients, although this difference was not statistically significant. Importantly, no early post-initiation relapse clustering was observed. These findings are consistent with large cohort studies and international consensus statements indicating that GH replacement does not increase recurrence risk when initiated after confirmed disease stability. Collectively, these findings reinforce current expert recommendations that GH therapy may be safely initiated in medulloblastoma survivors with stable disease after appropriate multidisciplinary evaluation [24,25,42,44,47].

### 4.5. Impact of Modern Treatment Era (2000–2023)

The study period spans a long treatment era with heterogeneous treatment practices and encompasses major advances in medulloblastoma management in recent years, including molecular subgroup stratification and the increasing use of proton therapy [6,18,48,49,50,51,52,53]. Although proton therapy may reduce radiation exposure to surrounding tissues, endocrine dysfunction remains common even with modern techniques [13,14,16,18,19,20]. Owing to the small number of patients with molecular subgroup data and those treated with proton (vs photon) therapy, risk stratification for GHD was not feasible in our study. The persistence of high GHD prevalence in our cohort highlights that, despite technological improvements, hypothalamic–pituitary vulnerability remains clinically relevant. Importantly, no differential safety signal related to treatment era was observed in GH-treated patients, supporting the applicability of GH replacement across contemporary oncologic protocols.

### 4.6. Clinical Implications

The present data emphasize several clinically relevant considerations. First, systematic endocrine surveillance is essential in medulloblastoma survivors given the high prevalence of GHD and other hormonal deficiencies. Second, untreated GHD results in progressive auxological deterioration and marked deviation from genetic height potential. Third, appropriately timed and monitored GH replacement therapy confers meaningful growth benefits without apparent increase in relapse risk. These findings support the integration of pediatric endocrinology into structured survivorship programs and reinforce the importance of multidisciplinary decision-making in GH initiation.

### 4.7. Strengths and Limitations

The strengths of this study include long-term follow-up exceeding a decade, the inclusion of patients treated in the modern era, the biochemical confirmation of GHD, and comparative analysis of treated and untreated patients. To the best of our knowledge, this is among the first studies to evaluate growth outcomes in pediatric medulloblastoma survivors, stratified by growth hormone deficiency status (no GHD, treated GHD, and untreated GHD). Nevertheless, several limitations should be acknowledged. The retrospective design introduces potential selection bias. The sample size, although consistent with single-center survivorship studies, limits statistical power for rare oncologic events. Endocrine assessments related to bone health, insulin resistance, and lipid profile were not available. This represents an important limitation, particularly for patients with untreated GHD. Molecular subgroup data were not uniformly available for all patients, which may influence risk stratification for growth impairment and relapse. Additionally, spinal growth impairment secondary to craniospinal irradiation may confound GH responsiveness. Future prospective multicenter studies with standardized GH initiation criteria and stratification by molecular subgroup and radiation modality are warranted.

## 5. Conclusions

In conclusion, in this cohort of medulloblastoma survivors diagnosed between 2000 and 2023, growth hormone deficiency was highly prevalent and associated with significant long-term growth impairment when untreated. GH replacement therapy resulted in significant improvement in height SDS and the mitigation of deviation from parental height potential. Importantly, no signal of relapse associated with GH therapy was observed in this limited cohort when treatment was initiated after stable remission; however, confirmation in larger cohorts is warranted. These findings align with current international evidence supporting the efficacy and oncologic safety of GH replacement in appropriately selected medulloblastoma survivors.

## Figures and Tables

**Table 1:** Demographic and clinical features of a pediatric cohort affected by medulloblastoma (*n* = 74 patients).

Demographic and Clinical Features		Total *n* = 74 Patients
Gender	Male	35 (47.3%)
	Female	39 (52.7%)
Age at diagnosis (years)		7.94 ± 0.56
Follow-up (years)		12.01 ± 0.52
Treatment	Surgery	71 (95.9%)
	Chemotherapy	70 (94.6%)
	Radiotherapy	67 (90.5%)
Endocrine disorders	Yes	64 (86.5%)
	No	10 (13.5%)
Growth Hormone Deficiency	Treated	23 (31.1%)
	Untreated	13 (17.6%)
	No	38 (51.3%)

**Table 2:** Baseline Anthropometric and Hormonal Data and Growth Hormone Treatment Characteristics of the Study Population.

		All (*n* = 74)	No GHD (*n* = 38)	GHD-Untreated (*n* = 13)	GHD-Treated (*n* = 23)	*p*
Age at medulloblastoma diagnosis (years)	Mean	7.94 ± 0.56	8.39 ± 0.75	7.1 ± 0.92	6.82 ± 0.66	0.3
	Median	8 (IQR 5–11)	9 (IQR 5.5–13)	8 (IQR 7–10.75)	6 (IQR 4–6.75)	-
Radiotherapy dose (Gy)	Tumoral Bed	**44.1 ± 12.01**	46.05 ± 3.31	42.34 ± 2.46	40.15 ± 3.64	0.5
	Craniospinal	**30.5 ± 8.01**	32.89 ± 2.16	30.74 ± 2.32	31.14 ± 1.64	0.45
Baseline H (SDS)		−2.2 ± 0.73	−2.17 ± 0.26	−2.14 ± 0.33	−2.25 ± 0.54	0.85
Puberty present at GH stimulation test		**-**	8/20 (40%)	4 (30.8%)	7 (43.8%)	0.98
Age at GHD stimulation test (years)	Mean	11.6 ± 1.52	11.9 ± 0.57	11.7 ± 1.02	10.5 ± 1.11	0.13
	Median	11 (IQR 8.5–12)	11 (IQR 9–14.5)	10 (IQR 9–13.75)	9 (IQR 6.5–9.5)	-
GH peak after arginine (ng/L)		6.4 ± 0.73	9.4 ± 1.01	1.83 ± 0.48	2.36 ± 0.48	<0.0001
GH peak after glucagon (ng/L)		6.9 ± 2.19	10.3 ± 0.18	2.52 ± 0.66	2.67 ± 0.56	<0.0001
CA–BA (years)		3.19 ± 1.73	2.58 ± 1.75	2.23 ± 0.19	3.49 ± 0.21	0.12
Years of GH treatment (years)	Mean	-	-	-	6.01 ± 3.24	-
	Median	-	-	-	6.5 (IQR 3.75–8)	-
Age at GHD treatment start (years)	Mean	-	-	-	10.9 ± 0.58	-
	Median	-	-	-	9 (IQR 6.75–10)	-
Mean GH dose (mg/kg/week)		-	-	-	0.17 ± 0.01	-
IGF during follow-up (ng/L)		241.4 ± 120.9	441.4 ± 120.9	156.6 ± 25.56	366.8 ± 27.12	<0.0001
H at last evaluation (SDS)		−1.27 ± 0.22	−0.78 ± 0.56	−1.97 ± 0.42	−1.72 ± 0.32	<0.0001
H at last evaluation-Baseline H (SDS)		−1.23 ± 0.16	0.59 ± 0.26	−1.93 ± 0.78	0.39 ± 0.06	<0.0001
Patients reaching FH		54 (73%)	28 (73.7%)	10 (76.9%)	16 (69.6%)	0.88
FH (SDS)		−1.54 ± 0.23	−0.68 ± 0.24	−2.45 ± 0.36	−1.71 ± 0.68	<0.0001
PH (SDS)		−0.05 ± 0.74	0.18 ± 0.53	−0.13 ± 0.16	0.038 ± 0.21	0.53
FH SDS-PH SDS		−1.63 ± 0.33	−0.77 ± 0.22	−2.57 ± 0.33	−1.73 ± 0.47	<0.0001
TSH deficiency		15 (20.3%)	3 (7.9%)	5 (38.5%)	7 (30.4%)	0.02
Primary hypothyroidism		26 (35.1%)	9 (23.7%)	8 (61.5%)	9 (39.1%)	0.04
Isolated hyperthyrotropinemia		24 (32.4%)	11 (28.9%)	7 (53.8%)	5 (21.7%)	0.12
Thyroid nodules		15 (20.3%)	7 (18.4%)	4 (30.8%)	4 (17.4%)	0.58
ACTH deficiency		12 (16.2%)	7 (18.4%)	2 (15.4%)	3 (13%)	0.75
Pubertal delay		15 (20.3%)	5 (13.2%)	3 (23.1%)	7 (30.4%)	0.33
Precocious puberty		6 (8.1%)	2 (5.3%)	1 (7.8%)	3 (13%)	0.55
Hypofertility		25 (33.8%)	12 (31.6%)	6 (46.2%)	7 (30.4%)	0.58

H—height; GH—Growth Hormone; CA—Chronologic Age; BA—Bone Age; FH—Final height; PH—Parental height; TSH—Thyroid-Stimulating Hormone; ACTH—Adrenocorticotropic Hormone.

**Table 3:** Relapse distribution among GH-treated and untreated patients.

Group	*n*	Relapses (*n*)	Relapse Rate	Median Follow-Up (Years)
GH-treated	23	2	8.7%	9
GHD-No therapy	13	2	15.4%	8
No GHD	38	5	13.2%	8

## Data Availability

Some or all datasets generated during and/or analyzed during the current study are not publicly available but are available from the corresponding author on reasonable request.

## Abbreviations

The following abbreviations are used in this manuscript:

CNS	Central Nervous System
GHD	Growth Hormone Deficiency
rhGH	Recombinant Human Growth Hormone
IGF-1	Insulin-like Growth Factor 1
SD	Standard Deviations
IQR	Interquartile
SDS	Standard Deviation Score
CA	Chronologic Age
BA	Bone Age
TSH	Thyroid Stimulating Hormone
ACTH	Adrenocorticotropic Hormone
RFS	Relapse-Free Survival
